# Direct-acting antivirals improve survival and recurrence rates after treatment of hepatocellular carcinoma within the Milan criteria

**DOI:** 10.1007/s00535-020-01747-y

**Published:** 2020-12-05

**Authors:** Hironori Ochi, Atsushi Hiraoka, Masashi Hirooka, Yohei Koizumi, Michiko Amano, Nobuaki Azemoto, Takao Watanabe, Osamu Yoshida, Yoshio Tokumoto, Toshie Mashiba, Tomoyuki Yokota, Masanori Abe, Kojiro Michitaka, Yoichi Hiasa, Kouji Joko

**Affiliations:** 1 Center for Liver-Biliary-Pancreatic Disease, Matsuyama Red Cross Hospital, Bunkyo-cho 1, Matsuyama, Ehime Japan; 2 Gastroenterology Center, Ehime Prefectural Central Hospital, Kasuga-cho 83, Matsuyama, Ehime Japan; 3 Department of Gastroenterology and Metabology, Ehime University Graduate School of Medicine, Shitsukawa, Toon, Ehime Japan

**Keywords:** Hepatocellular carcinoma, Direct-acting antivirals, Hepatitis C virus, Hepatic functional reserve

## Abstract

**Background:**

The effects of direct-acting antivirals (DAAs) on survival and recurrence rates after curative hepatocellular carcinoma (HCC) treatment in patients with hepatitis C virus (HCV) infection remain controversial.

**Methods:**

This retrospective, multicenter study involved Child–Pugh class A patients within the Milan criteria who had a first diagnosis of HCC and survived 6 months or longer after undergoing hepatectomy or radiofrequency ablation (RFA). The DAA-treated group (DAA group) included 56 patients, and the DAA-untreated group (untreated group) included 261 patients. The study was conducted using the propensity score-matched (1:2) DAA group and untreated group, 56 and 112 patients, respectively.

**Results:**

The survival rate at 48 months in the DAA group and the untreated group was 91.0% and 68.7%, respectively, showing significantly better survival in the DAA group (HR: 0.33; 95% CI 0.13–0.84; *p* = 0.021). The recurrence rate at 48 months was 36.7% and 66.7%, respectively, showing a significantly lower recurrence rate in the DAA group (HR, 0.46; 95% CI 0.27–0.77; *p* = 0.003). The median albumin–bilirubin (ALBI) score at 3 years post-HCC treatment was − 2.84 in the DAA group and − 2.34 in the untreated group. The ALBI score showed a significant improvement from baseline to 3 years post-HCC treatment (*p* = 0.001), whereas that in the untreated group showed a significant decline (*p* = 0.040).

**Conclusions:**

DAAs after HCC treatment prevents deterioration of hepatic functional reserve and significantly improves both recurrence and survival rates.

**Electronic supplementary material:**

The online version of this article (10.1007/s00535-020-01747-y) contains supplementary material, which is available to authorized users.

## Introduction

Hepatitis C virus (HCV) infection affects an estimated 1.8 million people globally and is the leading cause of hepatocellular carcinoma (HCC) [1]. Reports have indicated that the most serious outcome of HCV infection is hepatocarcinogenesis or progressive hepatic failure resulting in death [2], and that HCV infection increases the rate of complications associated with extrahepatic morbidity, which accounted for about 50% of the HCV deaths [3].

Interferon-free therapy with direct-acting antivirals (DAAs) has increased the rate of sustained virologic response (SVR) in all genotypes of HCV patients, with fewer adverse drug reactions [4–6].

Antiviral treatment has been reported to decrease the recurrence rate of HCC and improve the survival rate in patients who achieve SVR with interferon (IFN) therapy after HCC treatment [7–9]. On the other hand, there are reports of no suppression of HCC recurrence in patients who achieved SVR with IFN compared with patients without SVR [10, 11]. Conti et al. reported that DAA after HCC treatment did not suppress HCC recurrence in a group of DAA-treated patients, although it was a single-arm study conducted in that group alone [12], whereas Reig et al. reported that DAA use increased the risk of unexpected HCC recurrence [13]. However, there were no differences in the early HCC recurrence rate between patients who underwent IFN and those who underwent IFN-free DAA as antiviral therapies [14]. There are reports that DAA improves the survival rate, but not the recurrence rate [15, 16]. As these reports have shown, the HCC recurrence and survival rates after SVR achieved with DAA therapy remain controversial. There are few reports of which factors related to hepatic function affect the survival rate.

The present study was conducted in patients with and without DAA therapy after treatment for the first diagnosis of HCC at multiple institutions to analyze how DAA therapy affects post-HCC treatment survival and recurrence rates and hepatic functional reserve.

## Methods

In this retrospective study, all data of patients who underwent treatment for HCV-related HCC at 3 institutions (Matsuyama Red Cross Hospital, Ehime University Hospital, Ehime Prefectural Central Hospital) from January 2010 to December 2017 were collected. The diagnosis of HCC was either a pathological diagnosis or an imaging diagnosis with dynamic CT, Gd-EOB-DTPA-enhanced MRI, or contrast-enhanced ultrasonography using perflubutane, performed according to the guidelines of the Japan Society of Hepatology [17]. Each doctor made a clinical diagnosis of cirrhosis based on CT and US findings such as liver nodularity and blood chemical analysis in all patients. The subjects were patients with Child–Pugh class A at the time of HCC treatment, whose HCC was within the Milan criteria (singe lesion ≤ 5 cm or up to 3 lesions ≤ 3 cm each), and treated with radiofrequency ablation (RFA) or hepatectomy alone. Hepatectomy was selected if the maximum tumor diameter was ≥ 3 cm, but the final treatment method was selected with consideration for the location of tumors, age, comorbidities, etc. Both groups included only patients who had achieved a complete response (CR) by the modified response evaluation criteria in solid tumors (mRECIST) based on computed tomography (CT) or magnetic resonance imaging (MRI) within 1 month after RFA or hepatectomy [18].

Furthermore, patients in the DAA-treated group (DAA group) also met the following inclusion criteria: (1) first diagnosis of HCC; (2) absence of recurrence confirmed on dynamic CT or contrast-enhanced MRI within 1 month before receiving DAA therapy; (3) confirmed achievement of SVR12 after DAA therapy completion; (4) no IFN or other antiviral therapies to treat HCV before receiving DAA therapy; and (5) time from HCC treatment to DAA therapy of 365 days or shorter.

Patients in the DAA-untreated group (untreated group) met the following inclusion criteria: (1) first diagnosis of HCC and (2) no prior IFN, DAA, or other antiviral therapies to treat HCV.

In addition, patients with a first diagnosis of HCC that developed after DAA therapy and patients who failed to achieve SVR12 after DAA therapy were excluded from the DAA group. Patients whose observation period ended within 180 days after HCC therapy were excluded from both groups. The flow chart of this study is shown in Supplemental Fig. 1. Within the Milan criteria, there were 440 patients with initial hepatocellular carcinoma and Child–Pugh class A. Of these, 144 who received DAA therapy after HCC treatment, DAA administered after repeated HCC treatments (*N* = 64), and patients with more than 365 days from HCC treatment to DAA administration were excluded from the DAA group (*N* = 24). Patients with an observation period of ≤ 180 days (*N* = 35) were excluded from the untreated group (*N* = 296).

Imaging surveillance of post-treatment recurrence of HCC was conducted with dynamic CT, EOB-MRI, ultrasonography, or contrast-enhanced ultrasonography every 3 to 4 months, according to the guidelines of the Japan Society of Hepatology [17]. In post-treatment surveillance, AFP and DCP were used adjunctively with imaging tests, but the final diagnosis of recurrence was made by imaging diagnosis.

This study was approved by the institutional review board and ethics committee of Matsuyama Red Cross Hospital (No. 751). This study was registered with the UMIN Clinical Trials Registry as UMIN000038109. All medications were open-label drugs. All parts of the research were conducted in accordance with the principles in the Declaration of Helsinki.

### Data collection

The pre-HCC treatment imaging results and laboratory data saved as electronic medical records were collected. The categories of data were sex, age, platelets (PLT), albumin (Alb), total bilirubin (T.Bil), prothrombin time (PT), alanine aminotransferase (ALT), aspartate aminotransferase (AST), alpha-fetoprotein (AFP), maximum diameter of HCC lesions, and number of tumors. The fibrosis-4 (FIB-4) index was calculated as a serological marker of liver fibrosis, and the albumin–bilirubin (ALBI) score was calculated as an index of hepatic functional reserve [19, 20]. The post-treatment data on death/survival, cause of death, albumin and bilirubin levels, and ALBI score at 1 and 3 years post-HCC treatment were also collected. The day of HCC treatment was considered the first day of follow-up.

### Statistical analysis

First, categorical and continuous variables of the DAA-treated group (DAA group) and DAA-untreated group (untreated group) were analyzed by the Mann–Whitney U test and the chi-squared test, as appropriate. Before statistical comparisons of the survival and recurrence rates between the two groups, a propensity score was calculated using a logistic regression model to reduce bias in the pre-HCC treatment baseline characteristics between the two groups. The covariates were sex, age, PLT, Alb, T.Bil, PT, ALT, AST, AFP, maximum tumor diameter, and number of tumor lesions. Using the calculated propensity score, the ratio of the DAA group to the untreated group was matched at 1 to 2, respectively, with the absolute value of the difference in the propensity score set to 0.1. After matching, the HCC survival and recurrence rates were estimated using the Kaplan–Meier method, and the differences in survival and recurrence rates between the two groups were assessed by the log-rank test. In addition, the sensitivity of the recurrence rate assessments was evaluated by a Landmark analysis performed with exclusion of the patients with recurrence within 6 months or 9 months or 12 months.

Then, the factors contributing to prolonging survival time and time to recurrence were analyzed with a Cox proportional hazards model. Changes of albumin and bilirubin and albumin-bilirubin (ALBI) score from pre-HCC treatment to 1 and 3 years post-treatment were each compared, and the differences were analyzed by the Wilcoxon signed-rank test. In all statistical analyses, a *p* value < 0.05 was considered significant. All statistical analyses were performed with SPSS ver.23.

## Results

### Baseline characteristics

Table 1 shows the baseline characteristics of the 56 patients in the DAA group and the 261 patients in the untreated group who were included in the final data collected. The median age was 71 years in the DAA group and 75 years in the untreated group (*p* = 0.014). RFA was the most common treatment in both groups, with 44 patients (78.6%) in the DAA group and 215 patients (82.4%) in the untreated group receiving RFA. Hepatectomy was performed for 56% (*N* = 28) of tumors with a maximum diameter ≥ 3 cm (*N* = 50), and 8.9% (*N* = 24) of those < 3 cm (*N* = 267). The median FIB-4 index was not significantly different between the DAA and untreated groups, but the FIB-4 index was < 3.25 in 17.8% in the DAA group and 14.5% in the untreated group. The median time from HCC treatment to DAA therapy was 5.6 months. The median follow-up period was 40.0 months in the DAA-treated group and 35.9 months in the untreated group. Statistical analyses showed significant differences between the 2 groups in the baseline characteristics of age, ALT, and T.Bil.

**Table 1:** Patients’ baseline characteristics

Variable*	DAA (*n* = 56)	Untreated (*n* = 261)	*p* value
Sex (male:female)	31:25	157:104	0.459
Age	71 (56–87)	75 (46–91)	0.014
AST (IU/L)	56 (16–287)	49 (5–194)	0.297
ALT (IU/L)	46 (8–260)	40 (9–238)	0.049
PLT (10^4^/μL)	10.5 (1.9–30.2)	10.5 (1.6–35.9)	0.788
PT (%)	82.0 (59.3–113.1)	86.0 (44.5–132.0)	0.181
T.Bil (mg/dL)	0.9 (0.3–2.4)	0.7 (0.1–2.3)	0.033
Alb (g/dL)	3.9 (2.9–5.2)	3.8 (2.9–5.0)	0.117
FIB-4 index	5.69 (0.97–42.03)	5.37 (1.93–48.12)	0.574
ALBI score	− 2.58 (− 1.58–3.64)	− 2.50 (− 1.45–4.10)	0.444
Child–Pugh score			0.865
5	43	194	
6	13	67	
AFP (ng/mL)	15.0 (2.0–2191)	17.0 (1.0–2136)	0.813
Number of HCCs			0.326
1	38 (67.9%)	199 (76.2%)	
2	14 (25.0%)	53 (20.3%)	
3	4 (7.1%)	9 (3.4%)	
Diameter of tumor (mm)	18 (6–40)	20 (5–50)	0.512
HCC treatment			0.571
RFA	44 (78.6%)	215 (82.4%)	
Surgical resection	12 (21.4%)	46 (17.6%)	
DAA regimen			
Daclatasvir/asunaprevir	9 (16.1%)	N/A	
Ledipasvir/sofosbuvir	30 (53.6%)		
Ombitasvir/paritaprevir/ritonavir	4 (7.1%)		
Sofosbuvir/ribavirin	3 (5.4%)		
Elbasvir/grazoprevir	4 (7.1%)		
Glecaprevir/pibrentasvir	6 (10.7%)		
Months from HCC therapy to last observation	40.0 (9.6–60.5)	35.9 (6.5–112.3)	0.707
Months from HCC therapy to DAA	5.6 (1.6–11.4)	N/A	

*AST* aspartate aminotransferase, *ALT* alanine aminotransferase, *PLT* platelets, *PT* prothrombin time, *T.Bil* total bilirubin, *Alb* albumin, *FIB-4* fibrosis-4, *ALBI* albumin–bilirubin, *AFP* alpha-fetoprotein, *HCC* hepatocellular carcinoma, *DAA* direct-acting antiviral agent, *RFA* radiofrequency ablation

^*^Continuous data are presented as medians (min–max)

The propensity score-matched DAA group and untreated group included 56 and 112 patients, respectively (Table 2). There were no significant differences between the 2 groups in any of the pre-HCC treatment baseline characteristics. The FIB-4 index was < 3.25 in 17.8% of the DAA group and 16.1% of the untreated group.

**Table 2:** Patients’ baseline characteristics after propensity score matching

Variable*	DAA (*n* = 56)	Untreated (*n* = 112)	*p* value
Sex (male:female)	31:25	70:42	0.373
Age (y)	71 (56–87)	73 (50–91)	0.270
AST (IU/L)	56 (16–287)	53 (5–194)	0.874
ALT (IU/L)	46 (8–260)	44 (13–238)	0.675
PLT (10^4^/μL)	10.5 (1.9–30.2)	10.2 (2.8–35.9)	0.833
PT (%)	82.0 (59.3–113.1)	85.5 (55.8–132.0)	0.383
T.Bil (mg/dL)	0.9 (0.3–2.4)	0.8 (0.1–2.3)	0.468
Alb (g/dL)	3.9 (2.9–5.2)	3.8 (2.9–5.0)	0.634
FIB-4 index	5.28 (0.97–23.80)	5.86 (1.93–48.12)	0.565
ALBI score	− 2.58 (− 1.58–3.64)	− 2.52 (− 1.60–4.10)	0.710
Child–Pugh score			0.556
5	43	89	
6	13	23	
AFP (ng/mL)	15.0 (2.0–2191)	18.0 (1.0–3665)	0.714
Number of HCCs			0.882
1	38 (67.9%)	79 (70.5%)	
2	14 (25.0%)	27 (24.1%)	
3	4 (7.1%)	6 (5.4%)	
Tumor diameter (mm)	18 (6–40)	18 (7–49)	0.759
HCC treatment			0.393
RFA	44 (78.6%)	94 (83.9%)	
Surgical resection	12 (21.4%)	18 (16.1%)	
DAA regimen			
Daclatasvir/asunaprevir	9 (16.1%)	N/A	
Ledipasvir/sofosbuvir	30 (53.6%)		
Ombitasvir/paritaprevir/ritonavir	4 (7.1%)		
Sofosbuvir/ribavirin	3 (5.4%)		
Elbasvir/grazoprevir	4 (7.1%)		
Glecaprevir/pibrentasvir	6 (10.7%)		
Months from HCC therapy to last observation	40.0 (9.6–60.5)	43.2 (7.1–112.3)	0.090
Months from HCC therapy to DAA	5.6 (1.6–11.4)	N/A	
Cause of death			0.988
HCC	2 (50%)	22 (53%)	
Liver failure	1 (25%)	10 (24%)	
Extrahepatic disease	1 (25%)	9 (23%)	

^*^Continuous data are presented as medians (min–max)

*AST* aspartate aminotransferase, *ALT* alanine aminotransferase, *PLT* platelets, *PT* prothrombin time, *T.Bil* total bilirubin, *Alb* albumin, *FIB-4* fibrosis-4, *ALBI* albumin–bilirubin, *AFP* alpha-fetoprotein, *HCC* hepatocellular carcinoma, *DAA* direct-acting antiviral agent, *RFA* radiofrequency ablation

### Overall survival and the recurrence rate

Table 3 shows that DAA significantly improved the survival rate with the Cox proportional hazards model analysis (hazard ratio [HR], 0.33; 95% confidence interval [CI], 0.13–0.84; *p* = 0.021).

**Table 3:** Factors related to overall survival on Cox proportional hazards analysis

Variable	Category	Univariate analysis		
		HR	95% CI	*P* value
Age	≤ 69 years	1		
	≥ 70 years	1.329	0.73–2.40	0.346
Sex	Male	1		
	Female	1.212	0.68–2.14	0.508
AST	≤ 40 IU/L	1		
	≥ 41 IU/L	0.807	0.43–1.51	0.502
ALT	≤ 40 IU/L	1		
	≥ 41 IU/L	0.998	0.56–1.79	0.995
PLT	≥ 10(10^4^/μL)	1		
	≤ 9.9(10^4^/μL)	1.238	0.70–2.18	0.461
PT	≥ 80%	1		
	≤ 79.9%	1.107	0.62–1,97	0.732
T.Bil	≤ 1.9 mg/dL	1		
	≥ 2.0 mg/dL	0.779	0.19–3.22	0.731
Alb	≥ 3.6 g/dL	1		
	≤ 3.5 g/dL	1.686	0.85–3.34	0.134
Child–Pugh	5	1		
	6	1.353	0.70–2.60	0.821
AFP	≤ 10 ng/dL	1		
	≥ 11 ng/dL	1.421	0.74–2.74	0.294
Tumor size	≤ 29 mm	1		
	≥ 30 mm	1.402	0.70–2.83	0.344
Number of HCCs	1	1		
	≥ 2	1.705	0.96–3.04	0.071
DAA	No	1		
	Yes	0.33	0.13–0.84	0.021

*AST* aspartate aminotransferase, *ALT* alanine aminotransferase, *PLT* platelets, *PT* prothrombin time, *T.Bil* total bilirubin, *Alb* albumin, *AFP* alpha-fetoprotein, *HCC* hepatocellular carcinoma, *DAA* direct-acting antiviral agent

**Fig. 1:**
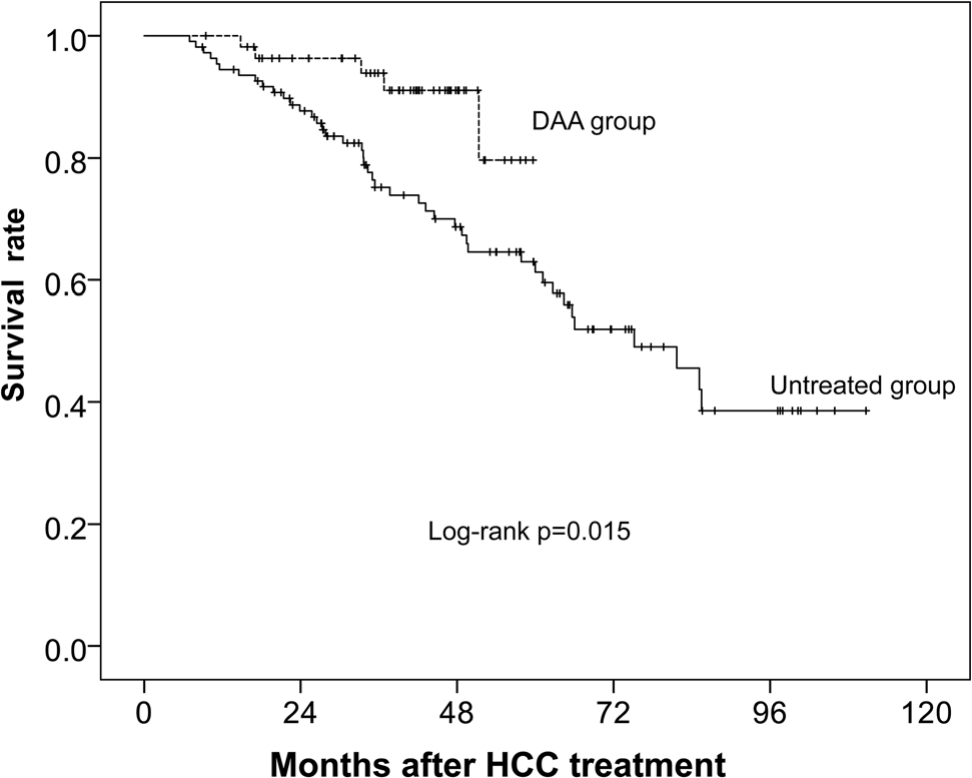
Survival curves after curative therapy of HCC in 56 patients given DAA therapy and 112 patients who did not receive DAA therapy. The solid line and the dotted line show the untreated group and the DAA group, respectively

Figure 1 shows the survival curves of the DAA and untreated groups after propensity score matching. The number of deaths during follow-up was 4 (7.1%) in the DAA group and 43 (38.3%) in the untreated group. The survival rates in the DAA and untreated groups were 98.2% and 94.5% at 12 months, 96.3% and 87.7% at 24 months, 93.9% and 75.2% at 36 months, and 91.0% and 68.7% at 48 months, respectively. Over the entire period, the survival rate was significantly better in the DAA group (log-rank test, *p* = 0.015). The mortality rate for every cause of death was lower in the DAA group than in the untreated group (Table 2).

Table 4 shows that DAA (HR, 0.46; 95% CI 0.22–0.77; *p* = 0.003) and tumor size (HR, 1.91; 95% CI 1.16–3.16; *p* = 0.012) were significantly associated with recurrence rate with the Cox proportional hazards model.

**Table 4:** Factors related to recurrence on Cox proportional hazards analysis

Variable	Category	Univariate analysis			Multivariate analysis		
		HR	95% CI	P value	HR	95% CI	P value
Age	≤ 69 years	1					
	≥ 70 years	1.07	0.70–1,64	0.087			
Sex	Male	1					
	Female	0.88	0.57–1.36	0.566			
AST	≤ 40 IU/L	1					
	≥ 41 IU/L	1.45	0.90–2.35	0.136			
ALT	≤ 40 IU/L	1					
	≥ 41 IU/L	1.29	0.84–1.98	0.254			
PLT	≥ 10 (10^4^/μL)	1					
	≤ 9.9 (10^4^/μL)	0.93	0.61–1.41	0.730			
PT	≥ 80%	1					
	≤ 79.9%	1.23	0.81–1.88	0.334			
T.Bil	≤ 1.9 mg/dL	1					
	≥ 2.0 mg/dL	0.38	0.92–1.56	0.173			
Alb	≥ 3.6 g/dL	1					
	≤ 3.5 g/dL	1.31	0.78–2.20	0.315			
Child–Pugh	5	1					
	6	1.34	0.83–2.18	0.231			
AFP	≤ 10 ng/dL	1					
	≥ 11 ng/dL	1.76	1.11–2.81	0.018	1.59	0.97–2.58	0.064
Tumor size	≤ 29 mm	1					
	≥ 30 mm	1.83	1.11–3.03	0.018	1.91	1.16–3.16	0.012
Number of HCC	1	1					
	2	1.66	1.08–2.56	0.021	1.48	0.97–2.32	0.086
DAA	No	1					
	Yes	0.49	0.29–0.81	0.006	0.46	0.27–0.77	0.003

*AST* aspartate aminotransferase, *ALT* alanine aminotransferase, *PLT* platelets, *PT* prothrombin time, *T.Bil* total bilirubin, *Alb* albumin, *AFP* alpha-fetoprotein, *HCC* hepatocellular carcinoma, *DAA* direct-acting antiviral agent

**Fig. 2:**
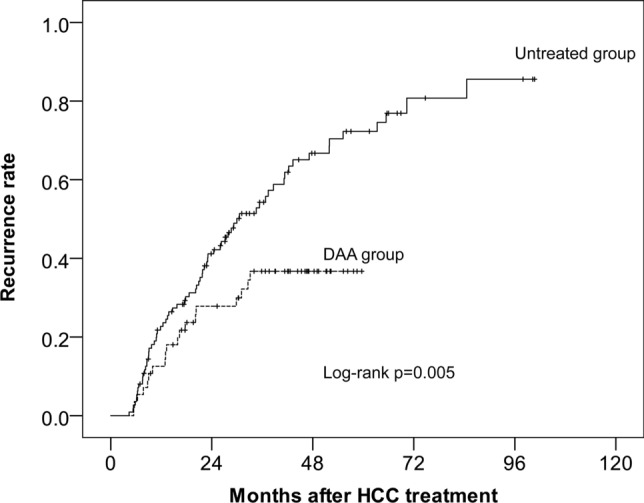
Cumulative incidence of HCC recurrence after curative therapy for HCC in 56 patients given DAA therapy and 112 patients who did not receive DAA therapy. The solid line and the dotted line show the untreated group and the DAA group, respectively

Figure 2 shows the recurrence curves during the follow-up period. The recurrence rate during follow-up was significantly lower in the DAA group than in the untreated group (log-rank test, *p* = 0.005).

The cumulative recurrence rate during follow-up was 33.9% in the DAA group and 62.5% in the untreated group. The recurrence rates in the DAA and untreated groups were 12.5% and 22.7% at 12 months, 27.8% and 41.1% at 24 months, 36.7% and 54.3% at 36 months, and 36.7% and 66.7% at 48 months, respectively.

**Fig. 3:**
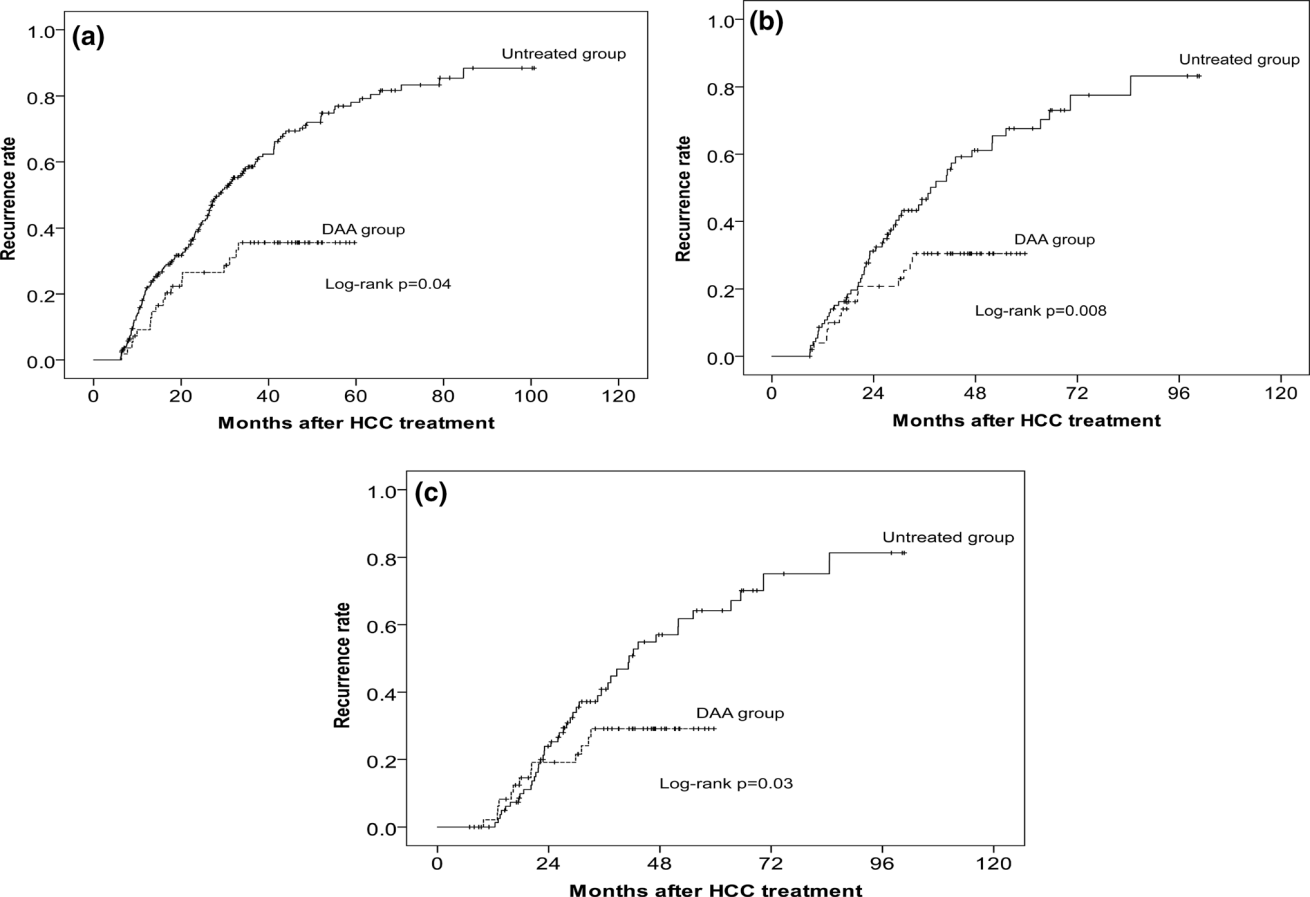
Landmark analysis with exclusion of patients who developed HCC recurrence within 3 months (**a**), 6 months (**b**), and 9 months (**c**) after curative therapy. Cumulative incidence of HCC recurrence. The solid line and the dotted line show the untreated group and the DAA group, respectively

Sensitivity was analyzed by a Landmark analysis performed with exclusion of patients who developed HCC recurrence within 6 months or 9 months or 12 months. The recurrence rate was significantly lower in the DAA group than in the untreated group (Fig. 3) (log-rank test: 3 months, *p* = 0.04; 9 months, *p* = 0.008; 12 months, *p* = 0.03).

### Classification of first recurrences and treatment options

In addition, first recurrences after HCC treatment were classified according to whether they were within or outside the Milan criteria (Supplemental Table 1). The first recurrence was outside the Milan criteria in 1 patient (5.1% of the recurred patients) in the DAA group and in 20 patients (39.2% of the recurred patients) in the untreated group, showing a significantly higher trend of recurrences classified as outside the Milan criteria in the untreated group (*p* = 0.036).

In both groups, all patients with recurrence within the Milan criteria chose to undergo RFA, whereas all patients with recurrence outside the Milan criteria received a non-curative treatment other than RFA or hepatectomy.

### Changes in albumin and bilirubin levels and ALBI score after HCC treatment

The median albumin levels in the DAA and untreated groups, respectively, were 4.1 (2.5–4.8) g/dL and 3.8 (2.6–4.3) g/dL after 1 year and 4.3 (2.2–4.7) g/dL and 3.7 (2.2–5.0) g/dL after 3 years. Although the change from baseline in albumin level was not significant at 3 years post-HCC treatment, the albumin level in the DAA group showed an improving trend (*p* = 0.068), whereas that in the untreated group showed a significant decline (*p* = 0.002).

The median bilirubin levels in the DAA and untreated groups were 0.8 (0.3–3.3) mg/dL and 0.8 (0.3–4.0) mg/dL after 1 year and 0.7 (0.4–1.8) mg/dL and 0.9 (0.8–3.0) mg/dL after 3 years, respectively. The bilirubin level at 3 years post-HCC treatment was unchanged from baseline in the DAA group, but it was significantly higher in the untreated group (*p* = 0.025).

The median ALBI score in the DAA and untreated groups were − 2.70 (− 1.28–3.30) and − 2.40 (− 1.24–4.26) at 1 year after HCC treatment and − 2.84 (− 1.16–3.33) and − 2.34 (− 0.86–-3.63) at 3 years after HCC treatment, respectively. The ALBI score in the DAA group showed a significant improvement from baseline to 3 years post-HCC treatment (*p* = 0.001), whereas that in the untreated group showed a significant decline (*p* = 0.040) (Supplemental Fig. 2).

## Discussion

The present study was a real-world observational study investigating the effects of DAA therapy on the prognosis and recurrence rate after curative HCC treatment only in patients with good hepatic functional reserve, classified as a Child–Pugh class A, who were within the Milan criteria. DAA use after HCC treatment was associated with improvement of both overall survival and the recurrence rate, while also maintaining hepatic functional reserve.

It has been reported that DAA use was associated with improvement of hepatic functional reserve, even in patients with poor hepatic functional reserve, such as those with decompensated cirrhosis [21, 22]. Given such obvious advantages of DAA use, it would be ethically difficult to conduct randomized, controlled trials (RCTs) between DAA and untreated groups. Thus, propensity score-matched assessments such as the present study are an effective means for evaluating the effects of DAA therapy. To the best of our knowledge, the present study is the first to assess the prognosis and recurrence rate after post-HCC treatment DAA use only in Child–Pugh class A patients within the Milan criteria.

An issue associated with all retrospective studies that examine the effect of drug treatment on survival, not just studies of DAA therapy, is the guarantee-time bias in favor of the treated group over the untreated group [23, 24]. Therefore, in the present study, only those patients who survived 6 months post-HCC treatment were included in either group before the propensity score matching. In addition, the time from HCC treatment to DAA therapy was limited to up to 12 months so as to avoid a bias in time to recurrence between the 2 groups.

Furthermore, sensitivity was assessed by a Landmark analysis at 6 months or 9 months or 12 months post-HCC treatment. In a retrospective, observational study comparing 2 groups, the Landmark method is useful for adjusting the bias over a span of time until the outcome under study occurs, as shown in the paper by Cho et al. [24]. Urania reported that the selection of landmark time creates a new bias [25]. If there is only one landmark time, the examination is an arbitrary analysis. Therefore, in the present study, three landmark times were selected to compare recurrence rates between the DAA and untreated groups. The recurrence rate was significantly lower in the DAA group.

The results of the present study showed that, most importantly, the overall survival rate was significantly better in the DAA group. The 5-year survival rate and the disease-free survival rate after hepatectomy and RFA for HCC within the Milan criteria have been reported to be approximately 76% and 54–61% compared to 40–50% and 20–30%, respectively [26–28], which is about the same as that in the untreated group in the present study, thus validating the population as a valid control group for the DAA group. One must also consider the recurrence pattern of the untreated group. Kamiyama et al. reported that 26.3% of hepatocellular carcinoma cases who underwent hepatectomy within the Milan criteria had recurrences outside the Milan criteria [29]. Lee et al. reported that 19.1% of hepatocellular carcinoma cases that underwent hepatectomy within the Milan criteria had recurrences outside the Milan criteria [30]. There are not many differences compared to the recurrence pattern of the untreated group in the present study. HCV-associated hepatocellular carcinoma tends to develop multicentric carcinogenesis more frequently than other causes of hepatocellular carcinoma [31, 32]. In other words, eradication of HCV by DAA suppresses multiple recurrences after HCC treatment. HCV-related HCC has different recurrence patterns depending on the time of recurrence after treatment. It has been reported that intrahepatic metastasis occurs within 2 years after treatment, and de novo recurrence occurs beyond 2 years [33, 34]. In particular, Imamura et al. reported that the degree of HCV hepatitis was associated with the cause of recurrence beyond 2 years after treatment [34]. In the present study, in the DAA group, the survival rate was better over the entire period, and the difference from the untreated group was particularly noticeable after 24 months, suggesting that DAA use after curative HCC treatment improves the mid to long-term prognosis. Significant improvement in the survival rate even after HCC treatment has also been reported in HCV patients who achieved SVR with IFN [35, 36]. Such improvements reflect the decreased hepatitis activity due to HCV eradication, which prevents deterioration of hepatic function and progression to hepatic failure. The present study also showed a very low probability of mortality due to hepatic failure during the follow-up, with only one such death among the 56 patients in the DAA group. The serum albumin and bilirubin concentration–time profiles were also compared between the two groups to investigate the actual changes over time in hepatic functional reserve. During the follow-up, the serum albumin level decreased in the untreated group, but it instead showed an increasing trend in the DAA group. Albumin levels are an important predictor of the prognosis of HCC and cirrhosis; high albumin levels are known to be associated with a better survival rate [37, 38]. Sugimoto et al. reported that DAA therapy increased albumin levels in HCV patients [39], but their study was conducted in patients without HCC. The present study showed that DAA therapy even after HCC treatment prevents deterioration of serum albumin levels. The ALBI score deteriorated in the untreated group 3 years after HCC treatment, but it improved significantly in the DAA group. The ALBI score is an index of liver reserve that includes only objective values. It has recently been reported that it is useful for predicting the prognosis of HCC after RFA or microwave ablation treatment and as an index of liver reserve during or after the introduction of tyrosine kinase inhibitor [40–42]. When SVR is obtained with DAA, hepatic reserve function is maintained, so that various curative treatment modalities can be used depending on the recurrence pattern. Extrahepatic mortality is said to account for about 50% of the deaths in patients with HCV infection [3, 43]. The present study also found that extrahepatic mortality was 8% in the untreated group compared to 1% overall in the DAA group, indicating that SVR achieved through DAA use lowers extrahepatic mortality. HCV is considered to cause many extrahepatic diseases, such as worsening insulin resistance, worsening cardiovascular conditions, and neurological diseases [44–46]. In a large prospective cohort study, HCV-RNA-positive patients were reported to have a lower mortality rate for non-liver diseases such as heart disease than HCV-RNA-negative patients [47]. In the present study, DAA reduced extrahepatic diseases. In addition, improved hepatic reserve function may reduce the probability of infection, bleeding disorder, and such.

The post-treatment recurrence rate in the present study was significantly lower in the DAA group than in the untreated group. DAA therapy does not completely suppress the development of hepatocellular carcinoma. If the FIB-4 index and AFP are high, it has been reported that HCC will develop in 14.6% of cases within 2 years in patients with no history of hepatocellular carcinoma [48]. Moreover, it is difficult for DAA therapy after HCC treatment to completely suppress recurrence. Two recent reports showed that the recurrence rate was unchanged in patients who received DAA therapy after HCC treatment compared with those in the untreated group [15, 16]. Differences in recurrence rates in the DAA group between the present study and the two reports are due to differences in tumor status and treatment at the time of HCC diagnosis and the observation period. Signal et al. reported that 19.3% of cases outside the Milan criteria were included, and treatment methods other than hepatectomy and treatment other than RFA accounted for 43.7% of the cases. In the study by Cabibbo et al., RFA and hepatectomy were selected as treatment methods, but the tumor diameter at the time of treatment was 2.44 cm, and AFP was 37.16 ng/mL; the tumor was larger than in the present study. In the present study, HCC was diagnosed at an early stage. This is because Japan has excellent surveillance for HCC. According to a report by Kudo, more than 60% of cases were diagnosed with Barcelona Clinic Liver Cancer stage 0 or A with Japanese surveillance [49]. In such cases, curative therapy such as hepatectomy or RFA can be performed, which contributes to improving the prognosis and reducing the recurrence rate. In other words, in patients with tumor lesions of smaller diameter and lower AFP levels who undergo curative treatment, DAA use may be more efficacious for suppressing recurrence, including unexpected recurrence, compared with DAA non-use. In the present study, there were no differences in recurrence rates between the two groups within an observation period of less than 20 months (Figs. 2 and 3). Since the effect of suppression of recurrence by DAA therapy appeared as a significant difference after about 20 months, the previous reports could not show the difference in the recurrence rate because of their short observation periods.

The present study, however, has some limitations. First, a large number of the patients underwent RFA, with no pathological examination performed for any of them. HCC was presumably diagnosed mainly on imaging with CT and MRI dynamic studies. However, recurrence and prognosis are said to be affected by the degree of tumor differentiation and vascular invasion, among other factors [50]. Such data were not investigated in the present study. Furthermore, the diagnosis of liver fibrosis was not confirmed histologically in most cases. We diagnosed cirrhosis based on clinical findings in all patients in the two groups. It would be more ideal if diagnoses could be made based on evaluation by elastography such as a Fibroscan. As the second-best measure, the FIB-4 index was calculated as a serological liver fibrosis marker. The cut-off value for cirrhosis is assumed to be a FIB-4 index > 3.25 [44]. In the present study, it was confirmed that there was no difference in the FIB-4 index between the two groups, but even after PS, about 17% of both groups had a FIB-4 index < 3.25, indicating that most cases were cirrhotic.

Second, the longer the time to treatment with the drug under study, the more susceptible survival and recurrence are to the effects of the issue of guarantee-time bias and other biases related to time, which often arise in non-RCTs. With the post-treatment follow-up period set to 180 days or longer as one of the inclusion criteria and the landmark method used to analyze the sensitivity of recurrence rate assessment, the effects of guarantee-time bias could be reduced, but not completely eliminated. Nevertheless, given that such an effect of time cannot be ruled out, we believe that it is necessary to collect data for further study in patients given DAA therapy with a shorter interval after HCC treatment.

The third limitation is that the differences between RFA systems were not considered. The start period of the untreated group in this study was mostly in the first half of the observation period. Another reason is that use of bipolar RFA started in January 2013, and the DAA group started almost at the same time in September 2014. However, bipolar RFA has also been reported to significantly reduce the recurrence rate in the same subsegment after treatment [51, 52]. Bias due to the difference between RFA devices in the two groups cannot be excluded.

In conclusion, in Child–Pugh class A patients with HCC within the Milan criteria, DAA use was very useful for improving the survival rate, decreasing the recurrence rate, and maintaining hepatic functional reserve.

## Electronic supplementary material

Below is the link to the electronic supplementary material.

Supplementary file1 (TIF 2176 KB)

Supplementary file2 (TIF 302 KB)

Supplementary file3 (DOCX 20 KB)
