# Supplementary figures and images for: Direct-acting antivirals improve survival and recurrence rates after treatment of hepatocellular carcinoma within the Milan criteria
Source: J Gastroenterol. 2020 Dec 5;56(1):90–100. doi: 10.1007/s00535-020-01747-y (PMC7819935; doi:10.1007/s00535-020-01747-y)

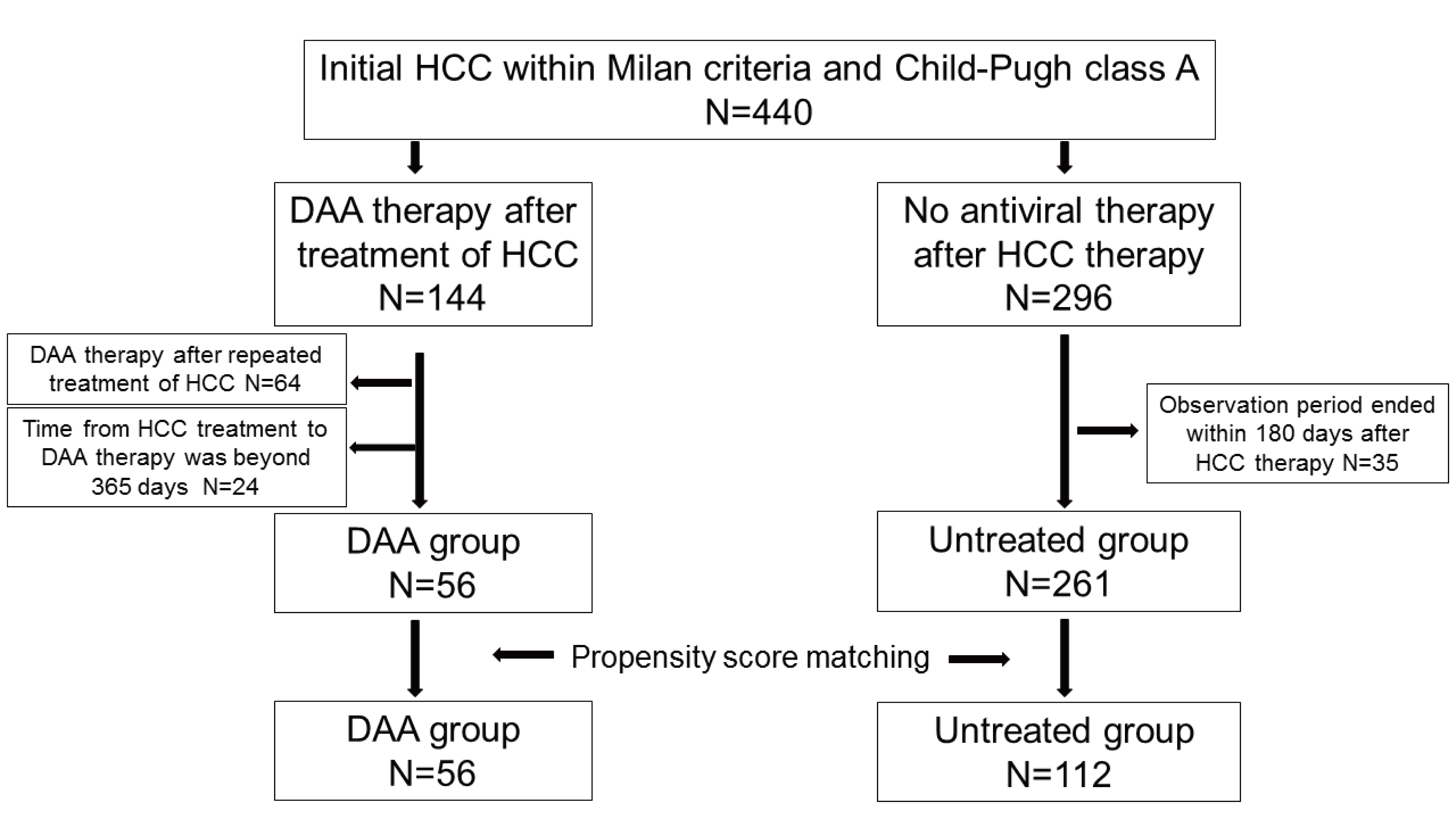

Supplement: Supplementary file 1 — Supplementary file1 (TIF 2176 KB) [file 535_2020_1747_MOESM1_ESM.tif]

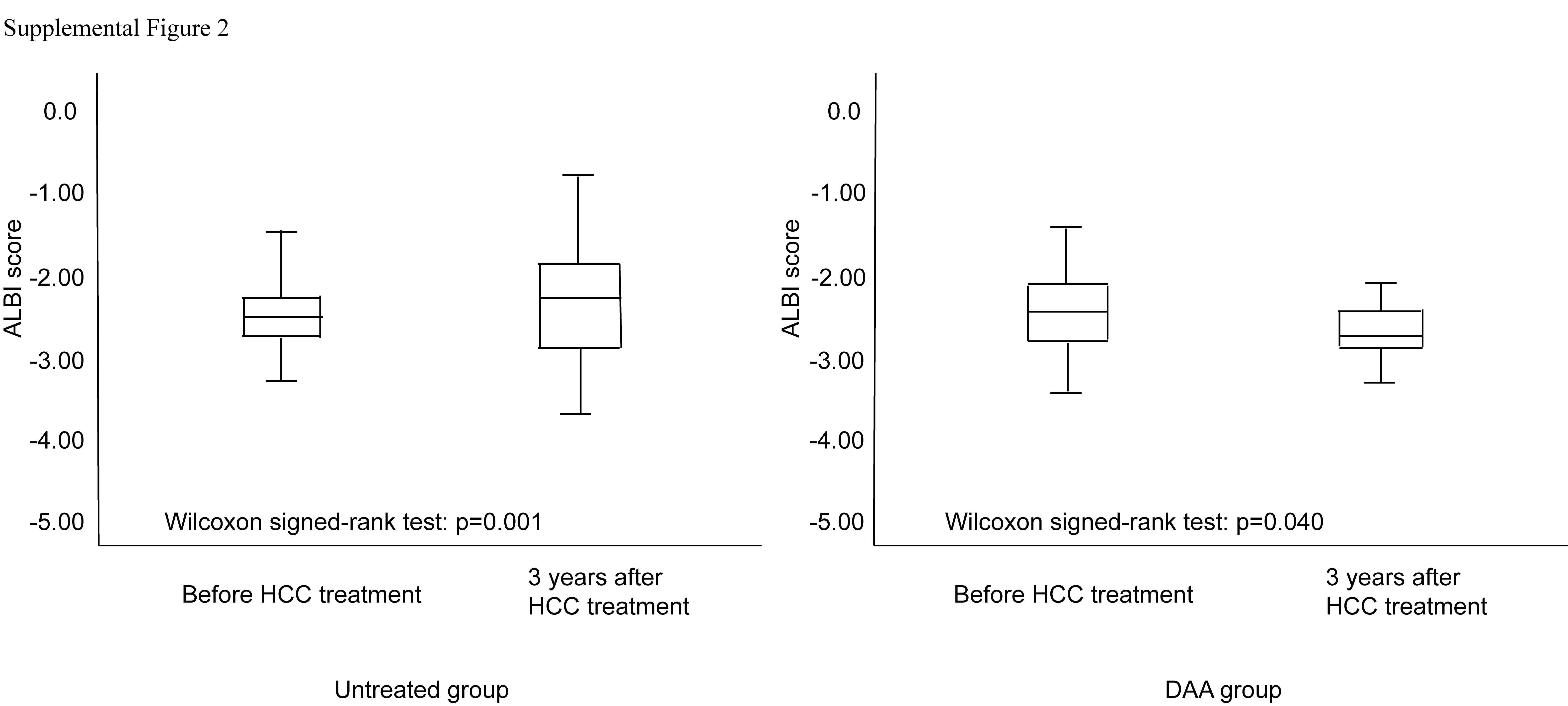

Supplement: Supplementary file 2 — Supplementary file2 (TIF 302 KB) [file 535_2020_1747_MOESM2_ESM.tif]
