# Supplementary material for: Direct-acting antivirals improve survival and recurrence rates after treatment of hepatocellular carcinoma within the Milan criteria
Source: J Gastroenterol. 2020 Dec 5;56(1):90–100. doi: 10.1007/s00535-020-01747-y (PMC7819935; doi:10.1007/s00535-020-01747-y)
Supplement: Supplementary file 3 — Supplementary file3 (DOCX 20 KB) [file 535_2020_1747_MOESM3_ESM.docx]

Supplemental Table 1. First recurrence patterns following HCC treatment after propensity score matching

|  | DAA (n=56) | Untreated (n=112) | p-value |
| --- | --- | --- | --- |
| Pattern of first HCC recurrence  Within Milan criteria  Outside Milan criteria | 18  1 | 51  20 | 0.036 |

AST, aspartate aminotransferase; ALT, alanine aminotransferase; PLT, platelets; PT, prothrombin time; T.Bil, total bilirubin; Alb, albumin; AFP, alpha-fetoprotein; HCC, hepatocellular carcinoma; DAA, direct-acting antiviral agent; RFA, radiofrequency ablation
